# Emotional dysregulation and Non Suicidal Self Injury in adolescents with ADHD transitioning to young adulthood

**DOI:** 10.1192/j.eurpsy.2025.156

**Published:** 2025-08-26

**Authors:** S. Marchini

**Affiliations:** 1 Faculté de Médecine, Université Libre de Bruxelles; 2 Service de Psychiatrie de l’Enfant et de l’Adolescent, Hôpital Universitaire de Bruxelles, Brussels, Belgium

## Abstract

**Abstract:**

Non-suicidal self-injury (NSSI) is a significant public health issue, particularly among adolescents with attention-deficit/hyperactivity disorder (ADHD). Characterized by inattention, hyperactivity, and impulsivity, ADHD is strongly linked to emotional dysregulation (ED) and psychiatric comorbidities, exacerbating vulnerability to self-injurious behaviors. This presentation examines the relationship between ADHD, ED, and NSSI, emphasizing clinical strategies for assessment and intervention during adolescence and the transition to adulthood.

Adolescents with ADHD often struggle with heightened emotional sensitivity and impaired emotion regulation, which can lead to NSSI as a maladaptive coping mechanism. ED mediates the connection between ADHD symptoms and NSSI. Evidence suggests that individuals with persistent ADHD and ED are at increased risk of psychiatric comorbidities such as depression, anxiety, and borderline personality disorder—factors independently associated with NSSI and suicidal behaviors (SB). Key findings include:

1. ED and impulsivity contribute to risk-taking behaviors, poor decision-making, and increased vulnerability to NSSI.
2. NSSI often begins in early adolescence and can escalate to severe SB, especially in individuals with co-occurring depression or adverse childhood experiences (ACE).
3. Girls with ADHD show higher rates of NSSI, often mediated by comorbid conditions such as depression and substance use disorders, underscoring the need for gender-specific interventions.

Cheng et al. (2024) demonstrated that ADHD subtypes marked by inattention were more associated with NSSI than hyperactivity/impulsivity, with anxiety as a significant mediator, especially for females. Ojala et al. (2022) found that childhood inattention predicts mid-adolescent NSSI, underscoring the importance of early detection. Balázs et al. (2018) highlighted the mediating roles of affective and psychotic disorders, with alcohol abuse uniquely influencing girls. Thornton et al. (2024) emphasized the role of cognitive disengagement syndrome (CDS) in predicting self-injurious behaviors.

These findings support integrating emotional regulation therapies into ADHD treatment frameworks. Interventions such as Dialectical Behavior Therapy (DBT), emotion-focused therapies, and ADHD medications addressing ED can mitigate risks of NSSI. Early identification and tailored care strategies targeting inattention and comorbid conditions are critical. Clinicians should screen for ADHD in adolescents presenting with NSSI and vice versa to ensure comprehensive treatment.

This presentation highlights the urgent need for developmentally sensitive approaches to mitigate NSSI risks in adolescents with ADHD, particularly during the vulnerable transition to adulthood. Addressing ED offers a promising avenue for reducing self-injury and improving psychosocial outcomes.

**Disclosure of Interest:**

None Declared

